# Effects of Long-Term Testosterone Therapy on Patients with “Diabesity”: Results of Observational Studies of Pooled Analyses in Obese Hypogonadal Men with Type 2 Diabetes

**DOI:** 10.1155/2014/683515

**Published:** 2014-03-11

**Authors:** Ahmad Haider, Aksam Yassin, Gheorghe Doros, Farid Saad

**Affiliations:** ^1^Private Urology Practice, 27570 Bremerhaven, Germany; ^2^Institute for Urology and Andrology, 22846 Norderstedt, Germany; ^3^International University, 01067 Dresden, Germany; ^4^Research Department, Gulf Medical University, Ajman, UAE; ^5^Department of Epidemiology and Statistics, Boston University School of Public Health, Boston, MA 02118, USA; ^6^Global Medical Affairs Andrology, Bayer Pharma, 13353 Berlin, Germany

## Abstract

To investigate effects of long-term testosterone (T) therapy in obese men with T deficiency (TD) and type 2 diabetes mellitus (T2DM), data were collected from two observational, prospective, and cumulative registry studies of 561 men with TD receiving T therapy for up to 6 years. A subgroup of obese hypogonadal men with T2DM was analyzed. Weight, height, waist circumference (WC), fasting blood glucose (FBG), glycated haemoglobin (HbA_1c_) blood pressure, lipid profile, C-reactive protein (CRP), and liver enzymes were measured. A total of 156 obese, diabetic men with T deficiency, aged 61.17 ± 6.18 years, fulfilled selection criteria. Subsequent to T therapy, WC decreased by 11.56 cm and weight declined by 17.49 kg (15.04%). Fasting glucose declined from 7.06 ± 1.74 to 5.59 ± 0.94 mmol/L (*P* < 0.0001 for all). HbA_1c_ decreased from 8.08 to 6.14%, with a mean change of 1.93%. Systolic and diastolic blood pressure, lipid profiles including total cholesterol: HDL ratio, CRP, and liver enzymes all improved (*P* < 0.0001). Long-term T therapy for up to 6 years resulted in significant and sustained improvements in weight, T2DM, and other cardiometabolic risk factors in obese, diabetic men with TD and this therapy may play an important role in the management of obesity and diabetes (*diabesity*) in men with T deficiency.

## 1. Introduction

Obesity has recently been recognized as a chronic disease condition necessitating appropriate medical treatment and not merely a transient condition that can be ameliorated simply with diet and exercise alone [1]. Obesity impacts quality of life and shortens life expectancy. A worldwide increase in the obesity epidemic has been reported. The increase in obesity, coupled with increased insulin resistance (IR) and T2DM, represents a healthcare crisis in developed and developing countries. Obesity is also associated with a host of other comorbidities including sleep apnoea, hypogonadism (testosterone deficiency, TD), dyslipidaemia, and hypertension. The prevalence of obesity and T2DM in the USA is higher than reported in other parts of the world and obesity is now considered a disease condition to be treated as any other disease conditions. The prevalence of diabetes increased by 7.3% and obesity increased by 7.8% during the past decade [2]. Obesity is associated with increased risk of IR and T2DM [3]. This increased risk was attributed to release of nonesterified fatty acids, glycerol, hormones, proinflammatory cytokines, and other factors from adipose tissue in obese individuals. The risk of developing T2DM may be reduced by 50–60% with a modest weight loss (WL) of 5–7% of body weight [4]. Huang et al. [5] projected the distribution of newly diagnosed, undiagnosed, and established cases of diabetes in the USA from 2009 to 2034 and estimated that the number of people with diagnosed and undiagnosed, diabetes will increase from 23.7 million to 44.1 million during this period. The obesity distribution in the population without diabetes will remain stable over time with ~65% of individuals of the population being overweight or obese [5]. Seidell et al. [6] reported that visceral fat accumulation is associated with increased insulin and C-peptide and with glucose intolerance [6].

In the early 1970s, Sims et al. [7] coined the term “diabesity” to describe the strong common link between diabetes and obesity, when they exist in the same individual. The risk of T2DM increases with body weight gain and obesity [8–12] and, more importantly, visceral fat accumulation reduces insulin sensitivity and increases IR, thus increasing risk of T2DM [13]. It is estimated that the risk of diabetes worldwide will exceed 171 million and may reach 366 million by the year 2030 [14]. Because increases in obesity are paralleled with increases in T2DM [14], the diagnosis of obesity and T2DM in the same individual presents clinical and therapeutic challenges to healthcare providers [15]. It should be recognized that T2DM complications contribute to cardiovascular disease (CVD), stroke, neuropathy, nephropathy, and retinopathy [16]. On the other hand, obesity confers increased hypertension, dyslipidaemia, stroke, cancer, depression, and obstructive sleep apnoea in addition to T2DM. Thus, the combination of T2DM and obesity in the same individual (diabesity) will have even more complications than either condition alone. The presence of obesity and T2DM in the same individual represents a complex relationship between these conditions. Clearly, it is established that the increased incidence of obesity and T2DM contributes to higher incidence of CVD, hypertension, stroke, cancer, and increased mortality [17, 18]. The frequency of CVD and T2DM increased with increased body mass index (BMI) and WC. This relationship between WC, CVD, and T2DM was noted even in patients with BMI ≤ 25 kg/m^2^ [17].

Recent studies have provided a critical assessment of the therapeutic options in diabetes and obesity and highlighted the various approaches used to date, including (a) antidiabetics, (b) incretin and glucagon like peptide-1 (GLP-1) receptor agonists and dipeptidyl peptidase inhibitors, (c) lifestyle modifications, (d) antiobesity agents, and (e) bariatric surgery [1, 19]. For instance, a recent report showed that bariatric surgery resulted in significant and sustained remission and improvement of T2DM and other metabolic factors in severely obese patients [20]. Indeed, some of the therapeutic strategies discussed above will be met with success in some patients while in others it may not. There remains a need for new and innovative alternative approaches to the management of diabetes and obesity. Recently, angiopoietin-like proteins (ANGPTLs) have been suggested as targets for treatment of obesity [21]. It was proposed that suppression of expression of ANGPTL2 and increased expression of ANGPTL6 may represent a therapeutic target for treatment of obesity [21].

Several studies have suggested that TD may contribute to development of obesity, IR, and T2DM and T therapy of men with TD may ameliorate these conditions [22–28]. Men with TD treated with T therapy experienced a positive effect on visceral obesity, as determined by reduction in body weight (−2.66%), waist-hip ratio (−3.96%), and body fat (−5.65%) when compared to the control group [29]. This treatment also resulted in decreased fasting blood glucose and mean glycated haemoglobin (HbA_1c_) [29]. Kapoor et al. [27] also demonstrated that T therapy in men with T2DM reduced visceral adiposity and reduced homoeostasis model assessment (HOMA) index, HbA_1c_, and fasting glucose, suggesting improvement in insulin sensitivity and glycaemic control in men with TD and T2DM [27]. Jones et al. [28] examined the effects of T therapy on IR, CVD risk, and symptoms of T deficiency in men with T2DM and/or MetS over a 12-month trial period. T therapy improved glycaemic control and reduced HOMA-IR as well as HbA_1c_, suggesting a beneficial effect of T therapy in men with T2DM [28]. In a study by Aversa et al. [24], in which the effects of T therapy on homoeostasis model assessment-estimated insulin resistance (HOMA-IR), carotid intima media thickness (CIMT), and high-sensitivity C-reactive protein (hsCRP) were investigated in men with TD and metabolic syndrome (MetS), T therapy significantly improved HOMA-IR, CIMT, and hsCRP as compared to the placebo treated group [24]. In another study in men with MetS, Kalinchenko et al. [30] found significant decreases in weight, BMI, WC, and HOMA-IR [30]. The effects of T therapy in 87 diabetic men with coronary artery disease (CAD) were investigated for a period of 12 weeks. T treatment significantly reduced the number of anginal attacks, silent ischaemic episodes, and total ischaemic burden, as compared with the placebo group. Total cholesterol (TC), plasma triglycerides (TG), and HOMA index were also significantly reduced in the T treated group compared to the placebo group, suggesting beneficial effects of T on T2DM complications [31]. In patients with chronic heart failure, T treatment significantly reduced insulin resistance measured by fasting insulin and HOMA-IR [32]. In a recent meta-analysis, Corona et al. [33] showed that T therapy was associated with marked reduction in fasting blood glucose, HOMA-IR, TG, and WC with concomitant increase in high density lipoprotein-cholesterol (HDL), suggesting that T therapy improves metabolic control and may ameliorate central obesity [33].

Several recent reports have shown that long-term T therapy in men with TD has produced significant weight loss, reduction in WC and BMI, as well as marked and significant reduction in total cholesterol, low density lipoprotein-cholesterol (LDL), TG, and increased HDL [23, 25, 34]. Also, marked reductions were noted in fasting glucose, HbA_1c_, the nonspecific inflammatory marker hsCRP, and liver enzymes aspartate aminotransferase (ASA) and alanine aminotransferase (ALA) suggesting improvement in hyperglycemia and reduction in the inflammatory response [23, 25, 34, 35]. The first controlled five-year study using T in men with MetS showed significant decreases in weight, WC, BMI, HbA_1c_, HOMA-IR, total cholesterol, LDL cholesterol, triglycerides, hsCRP, systolic, and diastolic blood pressure, and an increase in HDL [36]. In the current study, we present data on the long-term effects of T therapy in men with “diabesity.”

## 2. Methods and Procedures

This study represents a pooled subgroup analysis of obese hypogonadal men with T2DM from two cumulative registry studies of men, aged between 41 and 73 years (mean 61.17 ± 6.18), who were seeking urological consultation in two urologists' offices for various medical conditions such as erectile dysfunction, decreased libido, questions about their T status, or a variety of urological complaints. All subjects included in this study had subnormal plasma total T levels (mean: 8.9 ± 1.99; range: 1.63–11.79 nmol/L) and at least mild symptoms of hypogonadism assessed by the Aging Males' Symptoms scale (AMS). All patients had been diagnosed with T2DM prior to seeking urological consultation and treated accordingly by their family physicians with various standard treatment modalities. At their first visit, all men received brief general advice that it would be beneficial if they attempted to lose weight by a healthier diet consisting of more fruits and vegetables and less meat and increasing their physical activity by walking or using the bicycle instead of the car. They were not given any written instructions. All men received treatment with parenteral T undecanoate 1000 mg (Nebido, Bayer Pharma, Berlin, Germany), administered at baseline and 6 weeks and thereafter every 12 weeks for up to 72 months, as described previously [23, 25].

Measurements of anthropometric parameters (height, weight, and waist circumference) were performed and blood samples drawn at baseline and at the majority of visits prior to the next injection of testosterone. Therefore, T levels, measured by standard laboratory measurement, were trough levels at the end of an injection interval. All laboratory measurements for both centres were performed at the same commercial laboratory. Waist circumference (WC) was measured midpoint between the iliac crest and the lowest rib. Since not every measurement was performed at every single visit, values were averaged per patient and year.

Due to the cumulative registry design of the study, the number of subjects decreased over time. New subjects are entered into the database once they have received one year of treatment with T. All 156 subjects were followed for at least one year, 146 for at least two years, 136 for three years, 114 for four years, 105 for five years, and 69 for six years. The declining number of patients reflects duration of treatment but not the dropout rates. On the contrary, adherence to treatment was excellent, and T was only discontinued in two men who were diagnosed with prostate cancer.

## 3. Statistical Analyses

For continuous variables, the mean, median, standard deviation, range, minimum, maximum, and sample size for the overall sample and various groups were reported at each time point. For categorical variables the frequency distribution was reported. We tested the hypotheses regarding change in outcome scores across the study period by fitting a linear mixed effects model to the data. Time (to indicate follow-up interviews) was included as fixed effect in the model. A random effect was included in the model for the intercept. Estimation and test of change in scores were determined by computing the differences in least square means at baseline versus the score at each follow-up interview. For the correlation study, Pearson correlation was calculated between baseline changes in outcomes at various time points. The significance of each correlation was tested using Fisher's test.

## 4. Results

Table 1 provides the baseline characteristics for 156 obese, diabetic men with TD (mean age 61.17 ± 6.18 years). All 156 subjects had BMI ≥ 30 kg/m^2^. One hundred fifty-five men had WC ≥ 94 cm. Table 1 also contrasts the endpoints achieved with T therapy for most of the parameters described in the baseline characteristics.

The subjects in this study had several comorbidities. All 156 men had T2DM and dyslipidaemia, 153 men had hypertension, 37 men had a history of CAD, and 19 men had previously had a myocardial infarction. These are shown in Table 2. Table 2 also lists the concomitant medications related to T2DM, hypertension, and dyslipidaemia reported by the patients at baseline.

### 4.1. Total Testosterone Levels during the 6-Year Period of Testosterone Treatment

Total T levels showed a significant rise from 8.9 ± 1.99 nmol/L at the beginning of therapy to above 16 nmol/L within the first year of therapy, and such physiological levels remained constant at this level throughout the course of treatment, as reported previously [23, 25] (data not shown).

### 4.2. T Therapy of Obese Diabetic Men with TD Produced Reduction in Waist Circumference (WC)

**Figure 1:**
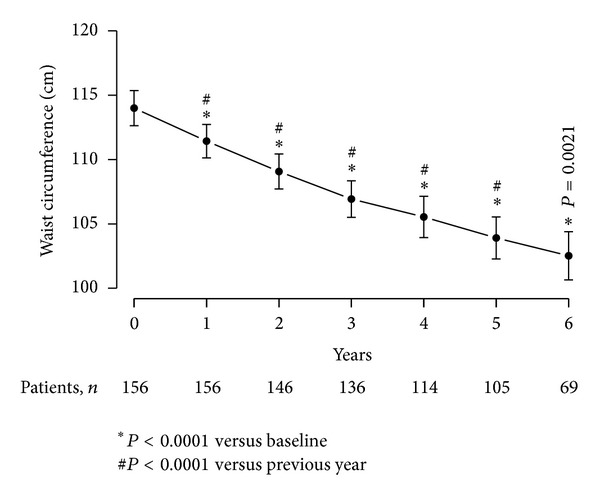
Waist circumference (cm) in 156 obese hypogonadal men with type 2 diabetes mellitus treated with testosterone undecanoate injections for up to 6 years.

Figure 1 demonstrates the measured reduction in WC subsequent to T therapy in obese diabetic men with TD. WC declined from 114 ± 8.69 cm (min 89, max 148) to 102.52 ± 7.93 cm (min 82.25, max 121) with a mean reduction of 11.56 ± 0.34 cm over the entire course of treatment (*P* < 0.0001). The reduction in WC was statistically significant at the end of each year compared to the previous year over the first five years (*P* < 0.0001) and had a statistical significance of *P* = 0.0021 at the end of six compared to five years. At the end of the observation period, 7 patients had achieved a WC below 94 cm.

### 4.3. T Therapy of Obese, Diabetic Men with TD Produced Significant Weight Loss (WL)

**Figure 2:**
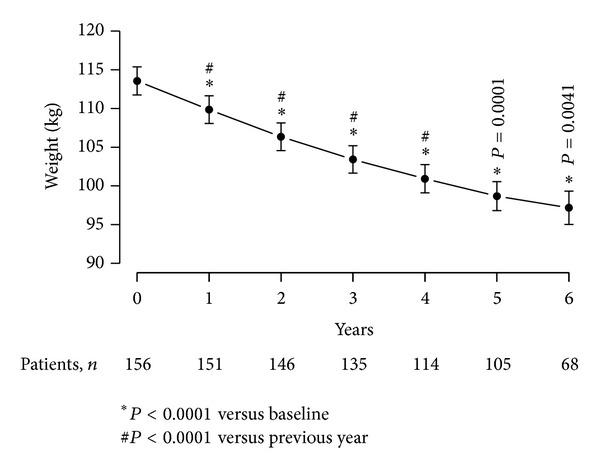
Weight (kg) in 156 obese hypogonadal men with type 2 diabetes mellitus treated with testosterone undecanoate injections for up to 6 years.

Figure 2 shows the effects of T therapy on the body weight of obese diabetic men with TD over the course of 6 years of therapy. Body weight decreased from 113.56 ± 11.53 kg (minimum: 87, maximum: 141) to 97.18 ± 9.04 kg (min 80, max 118.5) with a mean loss of 17.49 ± 0.58 kg over the course of treatment. This decrease in body weight was statistically significant at the end of each year compared to the previous year over the first five years (*P* < 0.0001) and had a statistical significance of *P* = 0.0041 at the end of six compared to five years.

### 4.4. Percentage Change in Body Weight as a Result of T Therapy of Obese Diabetic Men with TD

**Figure 3:**
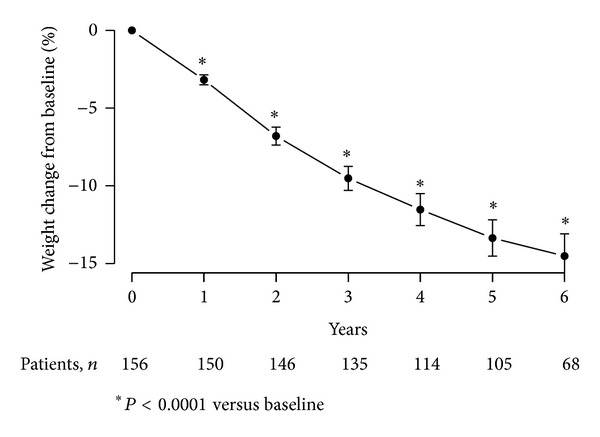
Weight change from baseline (%) in 156 obese hypogonadal men with type 2 diabetes mellitus treated with testosterone undecanoate injections for up to 6 years.

Marked and significant decrease in percentage body weight was noted over the course of T therapy. Over the entire 6-year observation period, patients lost 15.04% of their initial body weight (Figure 3). After one year, patients had lost 3.1 ± 0.37% of their initial weight, after two years, 6.82 ± 0.37%, after three years, 9.55 ± 0.38%, after four years, 11.78 ± 0.4%, after five years, 13.56 ± 0.41%, and after 6 years, 15.04 ± 0.48%. These changes were statistically significant versus baseline (*P* < 0.0001).

### 4.5. T Therapy of Obese, Diabetic Men with TD Produced Significant Decline in BMI

**Figure 4:**
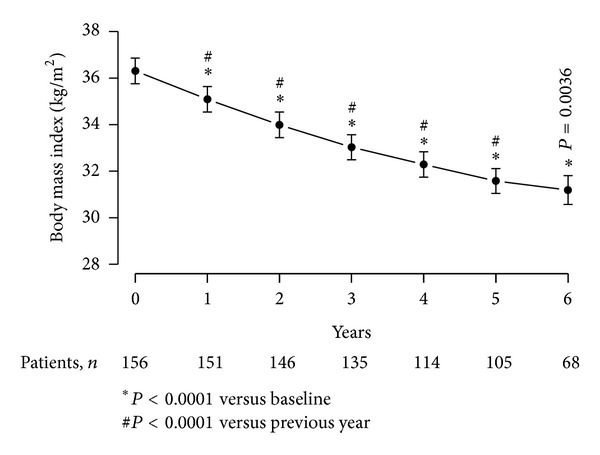
Body mass index (BMI, kg/m^2^) in 156 obese hypogonadal men with type 2 diabetes mellitus treated with testosterone undecanoate injections for up to 6 years.

Consistent and progressive decline in BMI was observed over the entire course of treatment with a mean reduction of 5.59 ± 0.18 kg/m^2^ (*P* < 0.0001) (Figure 4). BMI declined from 36.31 ± 3.51 to 35.09 ± 3.44 after one year, 33.99 ± 3.4 after two years, 33.03 ± 3.19 after three years, 32.29 ± 2.97 after four years, 31.58 ± 2.8 after five years, and 31.19 ± 2.6 after 6 years. The decline in BMI is consistent with the observed reductions in WC and body weight. At the end of the observation period, 48 patients (30.8%) were overweight and one patient (0.6%) had achieved normal weight.

### 4.6. T Therapy of Obese, Diabetic Men with TD Improved Blood Glucose Levels

**Figure 5:**
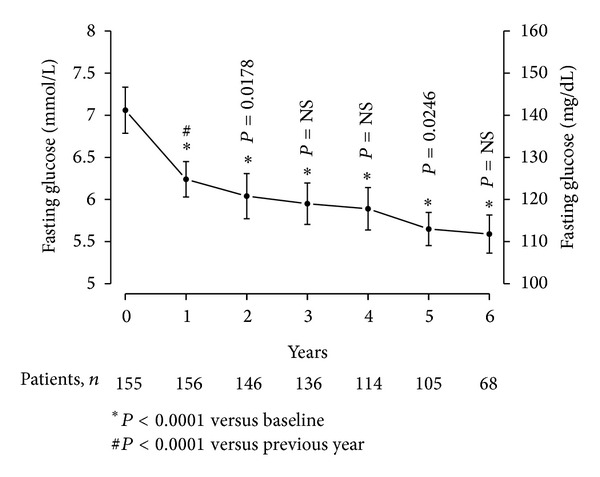
Fasting glucose (mg/dL or mmol/L) in 156 obese hypogonadal men with type 2 diabetes mellitus treated with testosterone undecanoate injections for up to 6 years.

T therapy of obese, diabetic men with TD resulted in a significant gradual decrease in fasting blood glucose from 7.06 ± 1.74 mmol/L (128.37 ± 31.63 mg/dL) to 5.59 ± 0.94 mmol/L (101.55 ± 17.02 mg/dL) (Figure 5). The decrease was significant after one year (*P* < 0.0001), further declined after two years (*P* = 0.0178 versus 12 months), and then reached a plateau with another slight but statistically significant decrease at five years compared to four years (*P* = 0.0246). A decrease of 1.49 ± 0.14 mmol/L (27.14 ± 2.48 mg/dL) over the course of six years treatment was noted.

### 4.7. Effects of T Treatment of Obese, Diabetic Men with TD on Haemoglobin *A*_1*c*_ (*HbA*_1*c*_) Levels

**Figure 6:**
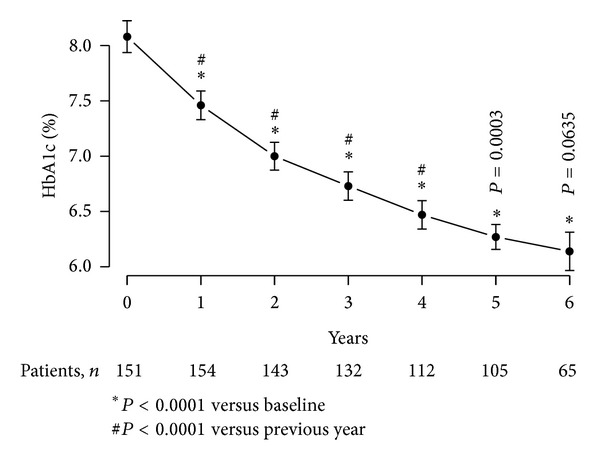
HbA_1c_ (%) in 156 obese hypogonadal men with type 2 diabetes treated with testosterone undecanoate injections for up to 6 years.

The decrease in fasting blood glucose was accompanied by a marked decrease in HbA_1c_ from 8.08 ± 0.09 % to 6.14 ± 0.71% with a mean change of 1.93 ± 0.06% (*P* < 0.0001) at the end of the observation period (Figure 6). The decrease in HbA_1c_ was progressive and statistically significant after one year (*P* < 0.0001), between two years and one year (*P* < 0.0001), between three and two years (*P* < 0.0001), between four and three years (*P* < 0.0001), and between five and four years (*P* = 0.0003) and approached significance between six and five years (*P* = 0.0635) (Figure 6).

**Figure 7:**
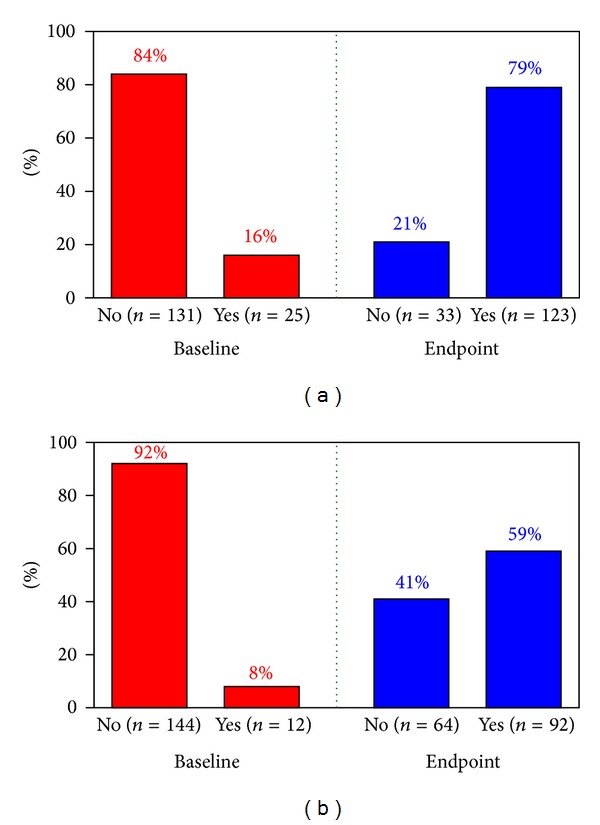
Patients reaching HbA_1c_ target of ≤7% (a) and ≤6.5% (b) at baseline and end of observation time after treatment with testosterone undecanoate injections for up to 6 years.

At baseline, 25 subjects (16%) had a HbA_1c_ target level of ≤7.0%. At the end of the observation period, 123 men (79%) had reached this goal (Figure 7(a)). At baseline, 12 patients (8%) had a HbA_1c_ level of ≤6.5%. At the end of the observation period, 92 men (59%) had achieved this target (Figure 7(b)).

### 4.8. T Therapy Improved Systolic and Diastolic Blood Pressure in Obese, Diabetic Men with TD

**Figure 8:**
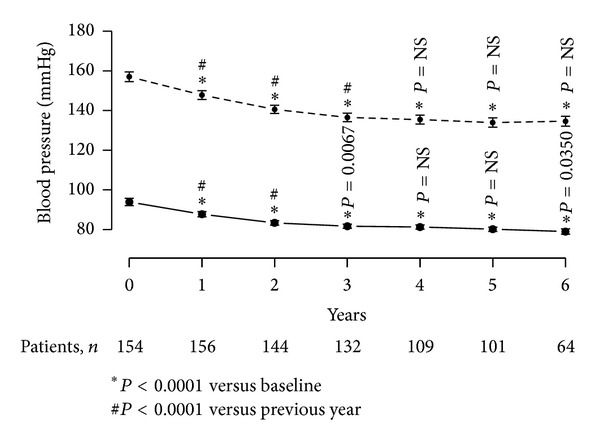
Systolic (dotted line) and diastolic (continuous line) blood pressure (mmHg) in 156 obese hypogonadal men with type 2 diabetes treated with testosterone undecanoate injections for up to 6 years.

T therapy of obese, diabetic men with TD produced marked and sustained gradual decrease in systolic blood pressure from 157.03 ± 15.46 mmHg to 134.61 ± 10.21 mmHg (*P* < 0.0001) over the course of 6 years of treatment. The mean decrease (23.15 ± 0.83 mmHg) was significant and progressive over the first three years and reached a plateau at this level over the remaining course of the 6 years of treatment. Similar results were recorded with the diastolic blood pressure which decreased from 93.89 ± 11.67 to 79 ± 5.57 mmHg (*P* < 0.0001) with a mean change of 15.07 ± 0.8 mmHg over the course of treatment (Figure 8). A gradual and progressive decrease was noted over the first three years of treatment and then blood pressure stabilized over the remaining years of treatment.

Another finding was a significant reduction of pulse pressure from 63.07 ± 10.7 (minimum: 35, maximum: 102) to 55.61 ± 8.62 (minimum: 38, maximum: 70). This decrease was statistically significant each year compared to the previous year during the first three years after which it remained stable.

### 4.9. T Therapy in Obese, Diabetic Men with TD Improved Lipid Profiles

**Figure 9:**
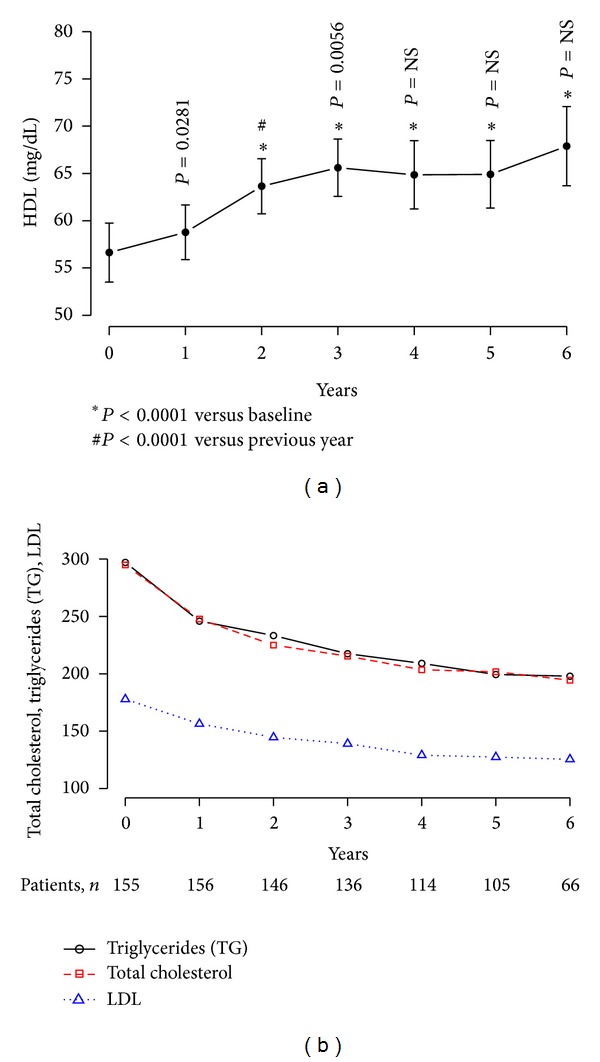
Changes in lipids (mg/dL) in 156 obese hypogonadal men with type 2 diabetes treated with testosterone undecanoate injections for up to 6 years. HDL cholesterol (a). Total cholesterol, LDL cholesterol, and triglycerides (b).

As shown in Figure 9, T therapy improved lipid profiles as demonstrated with increase in high density lipoprotein cholesterol (HDL-C) by 35.03 ± 5.11% (Figure 9(a)), significant reductions in total cholesterol (TC) by 32.12 ± 1.41%, low density lipoprotein cholesterol (LDL-C) by 25.93 ± 1.63%, and triglycerides (TG) by 29.91 ± 2% (Figure 9(b)). The mean changes in lipid profiles were gradual and progressive and were significant at each year when compared to baseline levels, reaching plateaus between three and four years. The ratio of total cholesterol to HDL cholesterol improved from 6.02 ± 2.97 to 3.05 ± 0.78. These changes reached a plateau after three years with further slight but not statistically significant decreases.

### 4.10. T Therapy in Obese Diabetic Men with TD Reduced the Levels of Inflammatory Biomarkers

**Figure 10:**
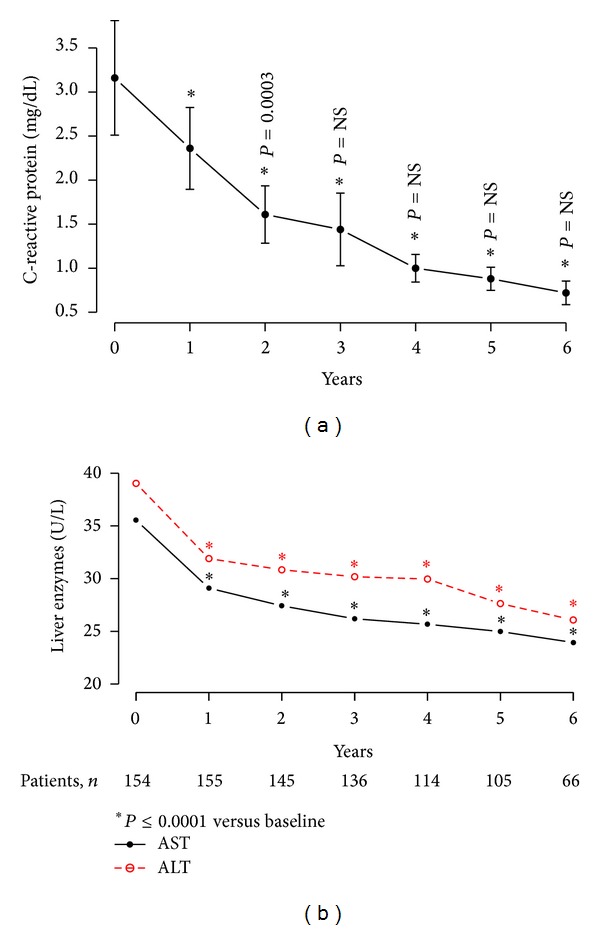
(a) C-reactive protein (mg/dL); (b) liver enzymes aspartate transaminase and alanine transaminase (U/L) in 156 obese hypogonadal men with type 2 diabetes treated with testosterone undecanoate injections for up to 6 years.

T therapy produced a marked and significant decrease in the concentration of the nonspecific inflammatory biomarker C-reactive protein (CRP) from 3.16 ± 4.12 to 0.72 ± 0.56 U/L (*P* < 0.0001 with a plateau after 24 months) and a significant (*P* < 0.0001) mean decrease of 2.88 ± 0.28 over the course of treatment (Figure 10(a)). Moreover, T therapy reduced the concentration of the liver enzyme aspartate transaminase (AST) from 35.55 ± 13.28 to 23.94 ± 8.77 U/L (*P* < 0.0001 with a plateau after 24 months) and a significant (*P* < 0.0001) mean change of 12.01 ± 1.33 U/L over the course of treatment. Similarly, alanine transaminase (ALT) concentration was reduced from 39.04 ± 19.38 to 26.08 ± 14.42 U/L (*P* < 0.0001) with a plateau after 12 months and significant mean change of 12.46 ± 1.83 U/L over the entire course of treatment, suggesting a reduction in liver fat content, a reduced inflammatory response, and improvement in liver function (Figure 10(b)).

### 4.11. T Therapy in Obese Diabetic Men with Total Testosterone below versus above 8 nmol/L

According to most of the current guidelines for testosterone replacement therapy, levels of total T below 8 nmol/L require treatment whilst levels above this threshold are considered borderline. We therefore analysed whether there were any differences in response in those of our patients who had T levels < 8 nmol/L versus those with T levels > 8 nmol/L and < 12 nmol/L. The results suggest that obese diabetic men in the higher T category respond to T treatment equally well (Table 3).

## 5. Discussion

In this long-term, cumulative, uncontrolled, and observational registry study from two independent sites, we investigated the effects of T therapy on anthropometric parameters, fasting blood glucose (FBG), HbA_1c_, systolic and diastolic blood pressure, lipid profiles, and inflammatory markers in 156 obese, diabetic men with TD. T therapy restored physiological T levels within the first 12 months and T levels were maintained with T therapy throughout the entire 6-year period. Of particular interest, our data demonstrate that T therapy in obese, diabetic men with TD produced significant and marked WL in 100% of all obese, diabetic patients. The WL subsequent to T therapy was gradual, progressive, and sustainable for the course of 6 years of treatment. The WL was significant and was associated with considerable and marked reductions in WC and BMI, suggesting that T therapy in obese, diabetic men results in improvement in metabolic function and probably behavioral changes with increased energy and motivation and physical activity that are translated into changes in body composition, which are accompanied by increases in lean body mass and reduction in fat mass, as discussed previously [22, 23, 25].

Over the entire observation period of 72 months, the longest follow-up reported to date, T therapy produced marked reductions in WC and BMI, consistent with the hypothesis that T is an anabolic hormone required for maintenance of muscle mass and regulation of adipogenesis [25]. It is of interest to note that the magnitude of changes over time in the obese, diabetic men was marked, progressive, and significant when compared with baseline data. These findings are not surprising since it is known that T promotes myogenesis and inhibits adipogenesis and regulates carbohydrate, lipid, and protein metabolism [25]. Thus, the changes in body composition noted in this study are attributed to T regulation of the metabolic processes in muscle and adipose tissues as well as functional metabolism.

It is important to point out that all these hypogonadal, obese men had been diagnosed with T2DM and received standard treatment by their family physician prior to initiation of T therapy. The mean baseline for HbA_1c_ in this study was 8.08% and only 25 men had an HbA_1c_ ≤ 7.0%, suggesting an overall poor control prior to T therapy. Interestingly, however, T therapy produced marked decrease in fasting blood glucose and HbA_1c_, suggesting that T therapy ameliorates hyperglycemia and IR in subjects with T2DM, consistent with previous reports [25]. These findings are also congruent with those reported in the IPASS study [37] in which 1438 men with TD were treated with T and followed up for up to 12 months suggesting similar reductions in glucose and HbA_1c_, particularly in patients with a poor HbA_1c_ control [37].

We also noted a meaningful reduction in systolic and diastolic blood pressure in response to T therapy. That T therapy improves hypertension in patients with TD is of interest, but limited data are available. It is possible that T modulates arterial blood pressure, through a variety of mechanisms, such as direct effects on the heart, the kidney, and the vessels, as well as the endothelium [38, 39]. Our data are consistent with previous work in obese, hypogonadal, and diabetic men in which blood pressure was shown to decrease favorably in response to therapy with an oral T formulation [29, 40].

Men with prostate cancer who were treated with androgen deprivation therapy (ADT) showed increased arterial stiffness [41, 42]. In our study, we observed a significant decrease of pulse pressure. Elevated pulse pressure is associated with an increase in large artery stiffness and recognized as an independent risk factor for CVD [43]. Our results are consistent with a placebo-controlled study that demonstrated a reduction in arterial stiffness, measured by use of the augmentation index, following T therapy [44].

CRP, a nonspecific marker of inflammation, was markedly reduced over the course of T therapy in obese, diabetic men with TD. Since it has been shown that WL significantly reduces plasma C-reactive protein (CRP) concentration [45], it is likely that the reduced weight reestablishes a new equilibrium with attenuated inflammatory responses and reduced CRP levels. It has been suggested that CRP concentration was significantly and directly associated with change in systolic blood pressure (SBP) and WC but inversely associated with HDL cholesterol [46]. Since T therapy improved both systolic blood pressure and reduced WC in this cohort of obese, diabetic men, it is not surprising that CRP levels were significantly reduced.

In addition, we noted a marked reduction in the activities of several liver enzymes, used as markers of nonalcoholic fatty liver disease (NAFLD) and liver function, suggesting that T therapy reduces liver fat content and attenuates the inflammatory response and improves various physiological functions. Our findings are consistent with the work by Hoyos et al. [44] who showed a reduction in liver fat content assessed by diagnostic imaging [44]. Taken together, these findings strongly suggest that normalizing T levels in obese men with TD ameliorates a host of MetS components and reduces inflammation, thus reducing the risk of cardiometabolic diseases.

It is of interest to note that, in this group of obese, diabetic men with TD, T therapy markedly and significantly reduced total cholesterol (TC) levels and this diminution was very pronounced and sustained over the entire 6-year period of T treatment. Further, we noted that long-term T therapy reduced LDL throughout the treatment period, and LDL levels were maintained at low levels throughout the course of treatment. The clinical implication of this observation is that reduction in LDL correlates with reduced CVD risk. Further, T therapy not only reduced the levels of LDL and TC but also produced small yet significant increases in HDL levels. Moreover, the ratio of total cholesterol to HDL cholesterol dropped from >6 to <3.5. These findings suggest that T therapy of obese, diabetic men with TD may reduce CVD risk and increase health benefits, such as improved lipid profiles. Another important observation in this study was the marked and significant reduction in triglycerides (TGs) in response to T therapy in these obese, diabetic men with TD. Since visceral fat storage depends on accumulation of TGs, thus, in men with TD, it is expected that increased lipid accumulation will contribute to obesity and T therapy is expected to produce reduction in body weight, WC, and BMI [23, 25]. Finally, a closer look at the per cent reduction in the lipids concentrations (TC, LDL, and TGs) indicated that the reductions approach 30%, a value similar to that attained by use of statins in men with dyslipidaemia. This is an important finding and warrants further investigations.

Obesity is a public health threat and its prevalence continues to increase worldwide [44, 47]. Obesity is associated with numerous comorbidities including T2DM, hypertension, stroke, and coronary artery disease [48]. From a global health policy perspective, the increased prevalence of T2DM and obesity epidemics represents a warning salvo for all healthcare systems. Evidence is accumulating suggesting that WL improves insulin sensitivity and *β*-cell function [49, 50]. It has been suggested that a loss of approximately 5–7% of body weight reduces the risk of developing diabetes by roughly 58% [4, 51]. The potential benefits of a moderate 5–10% WL in high risk patients with a cluster of atherothrombotic proinflammatory abnormalities associated with hypertriglyceridaemic waist have been proposed by Després et al. [52]. The authors suggested that visceral obesity leads to T2DM, dyslipidaemia, and hypertension, which result in increased risk and development of CVD, and a 5–10% WL can be very beneficial for improving health status and this should be pursued by behavioral therapy, exercise, diet, and pharmacotherapy [52].

The role of obesity in development of TD, T2DM, and inflammation is complex and involves a bidirectional and integrated variety of endocrine factors that results in increased IR, decreased T secretion, and increased inflammatory cytokines [53–56]. Increased levels of circulating free fatty acids and blood glucose result in triglyceride formation and storage, thus increasing visceral and subcutaneous fat mass. This further promotes additional release of free fatty acids into the circulation due to activation of lipases. Increased glucose is taken up by the liver. The increased sugar and fat uptake by the liver impairs the ability of insulin to regulate gluconeogenesis and activate glycogen synthesis. Inflammatory cytokine expression is increased which contributes to the development of insulin resistance. A recent study by Phillips and Perry [57] suggested that reduced inflammatory status increases the likelihood of metabolic health, particularly among obese subjects. Schnell et al. [58] showed that treatment which reduced inflammation, as assessed by CRP levels, was also associated with reductions in HbA_1c_ in diabetic individuals. Most studies have shown that HbA_1c_ control is not sustainable [59, 60]. However, we observed that T therapy produced significant and sustained reduction in HbA_1c_, suggesting that normalizing T levels reduces inflammation and restores glycaemic control [25].

The prevalence of T2DM is attributed, in part, to the increased prevalence of obesity combined with aging [14, 61]. The increased prevalence of obesity together with diabetes in the same individuals, henceforth referred to as “diabesity,” has also been reported, and individuals with “diabesity” exhibited higher risks of CVD [62, 63]. Obesity is also associated with increased expression of inflammatory cytokines. These inflammatory cytokines contribute to the impairment of insulin action and increased IR [64]. In a recent study of middle-aged individuals without glucose lowering medication the contributions of IR and hyperglycaemia to the development of subclinical atherosclerosis were attributed to a large extent to abdominal adiposity [65]. These findings suggest that weight management and prevention of weight gain in adulthood are critical for management of IR, obesity, and CVD. Casanueva et al. [18] reported that abdominal obesity was strongly associated with CVD and diabetes, even in patients who may be considered lean based on BMI assessment.

It is well recognized that adipose tissue is a unique endocrine organ which produces proinflammatory cytokines and free fatty acids that may promote development of IR and therefore will contribute to the development of atherosclerosis and CVD through hyperglycemia, inflammation, dyslipidaemia, and hypertension [65–67]. We believe that T therapy may confer health benefits since it results in significant and sustained WL in individuals with abdominal adiposity, as suggested previously [68]. One of the key measures of WL is the reduction in WC [69]. In fact, some studies have suggested that WC may be a risk factor for all-cause mortality [69, 70], incident CVD, and diabetes [17, 71]. Tseng [72] suggested that BMI and WC may be used as determinants of CAD in men with T2DM. Since T therapy produces significant reductions in WC, we believe that T therapy may provide significant health benefits in obese and diabetic patients with TD and may improve cardiometabolic function and reduce the risk of CVD.

Therapeutic approaches to treatment of obesity and diabetes include bariatric surgery, which were reported to be successful in management of this condition, producing rapid and durable weight loss, reductions in CVD events and overall mortality, and sustained remission of diabetes in most patients [73, 74]. However, bariatric surgery is not indicated for all obese diabetic patients and only select patients undergo this procedure, and thus this may be considered to be a limitation, given the large number of obese/diabetic patients.

Several other therapeutic approaches have also been used as potential treatment for diabetes, including sulfonylureas, thiazolidinediones, metformin, and insulin. However, with the exception of metformin, many of these agents result in weight gain and may increase IR risk. Alternative approaches also include the development of stable glucagon like peptide 1 (GLP-1) receptor agonists, which increase insulin secretion and suppress glucagon secretion and appetite [75]. GLP-1 is an effective agent in improving glycaemic control, when administered subcutaneously in patients with T2DM [76, 77]. Also the use of dipeptidyl peptidase inhibitors (DPP-4) to increase the intracellular level of endogenous GLP-1 has been proposed [78]. DPP4 inhibitors do not cause WL but instead are “weight neutral” [78]. These approaches were considered advantageous in treating diabetes when compared with the thiazolidinediones or sulfonylureas which result in weight gain.

T is well known to regulate a host of metabolic functions in liver, adipose tissue, muscles, coronary arteries, and the heart. Thus, it is not surprising that T therapy reduces the risk of CVD. An inverse relationship exists between T and obesity and TD is associated with dyslipidaemia, atherosclerosis, cardiovascular diseases, MetS, and diabetes (for review cf. Kelly et al. [54–56]). Several studies have suggested an association between reduced T levels and visceral obesity and diabetes. Visceral fat accumulation in men was positively associated with IR, hyperglycaemia, and C-peptide and is negatively associated with T levels [6]. The proportion of men with TD was increased in men with T2DM [79]. A significant association between TD and BMI and WC was noted in 355 male patients with T2DM (mean age 58 years) [27]. These findings suggest that WC and BMI in diabetic patients are associated with increased obesity. These findings are further supported by the work of Hackett et al. [35].

A number of studies have shown that T therapy in diabetic men with TD reduced body weight, WC, and body fat and reduced fasting blood glucose and HbA1c [24, 27–33]. These findings support the data presented in this study and suggest that T therapy may be a novel therapeutic approach to the management of T2DM and obesity in men with “diabesity.”

It should be pointed out that a number of studies have evaluated several drugs approved for management of diabetes and obesity with some positive outcome, albeit the efficacy of these drugs in maintaining WL or sustained weight loss remains debatable. Dyson [80] summarized the findings from studies on lifestyle, diet, and behavioral interventions on WL and WL maintenance. Based on a systematic review and meta-analysis, the authors suggested that all lifestyle interventions have a modest but significant effect on WL ranging between 1 and 4.9 kg in the overweight and obese subjects [80]. The authors concluded that exercise alone results in a moderate effect but is more effective when combined with dietary interventions. It was strongly suggested that behavioral therapy is an effective approach when combined with diet and exercise [80]. The diabetes prevention program (DPP) research group investigated the long-term safety and tolerability of metformin, along with WL, and change in WC during the diabetic prevention program (DPP) in a randomized double-blind clinical trial followed by a 7-8-year open-label extension [81]. Metformin treatment resulted in modest WL and WC, when compared with placebo. Most importantly, the magnitude of WL during the 2-year period was dependent on patients' adherence to treatment. During the open follow-up period, WL remained significantly greater in the metformin group than in the placebo group. In a double-blind clinical trial, Smith et al. [82] investigated the effects of lorcaserin together with diet and exercise and counseling in a large number of obese or overweight adults over a period of 52 weeks. After one year of treatment, approximately 47.5% of patients who received lorcaserin lost 5% or more of their body weight (~5.8 ± 0.2 kg) [82]. Interestingly, even in the treatment group, some weight gain was noted over the course of the second year of treatment. When treatment was discontinued, patients regained the weight lost over the course of the year. Gadde et al. [83] investigated the effects of oral phentermine and topiramate on WL in overweight or obese patients with several comorbidities such as hypertension, dyslipidaemia, diabetes, or abdominal obesity. The authors suggested that the combination of phentermine and topiramate, with office-based lifestyle interventions, might be a valuable treatment for obesity. In a subanalysis of the aforementioned study, the investigators could show reductions of 70.5% and 78.7% in the annualised incidence rate of T2DM [84]. Astrup et al. [63] investigated the effects of liraglutide over a two-year period and assessed the effects of this drug when coupled with diet and exercise on mean change in BW and WC in obese patients. The findings of this study indicated that liraglutide, together with diet and exercise, provides sustained WL over 2 years, with improvements in obesity associated metabolic and cardiovascular risk factors [63]. Chilton and colleagues [68] analysed data comparing orlistat or lorcaserin to lifestyle modifications (placebo) with sibutramine, rimonabant, or metformin on changes in WC and dropout rates attributed to adverse events of these agents. The authors suggested that orlistat significantly reduced WC by 6.96 cm when compared to placebo at 6 and 12 months. Lorcaserin also reduced WC and was more effective than that of other interventions at 12 months. Approximately 6.5% of patients on orlistat and 5.4% of those on lorcaserin discontinued their treatment due to adverse events at 12 months. The authors suggested that orlistat should be combined with lifestyle interventions in the treatment of obesity.

Thus, while a number of drugs, combined with behavioral and lifestyle changes, are shown to have moderate effect on WL and maintenance of weight loss, our data suggest that T therapy in obese diabetic men has a profound effect on WL and WC and this is irrespective of diet and exercise or behavioral counseling. This is meaningful, since the WL and the reduction in WC were observed over a long period of followup. We believe that since obesity is associated with hypogonadism (TD) and may, in fact, cause TD, long-term T treatment in hypogonadal men results in continuous improvements in weight and WC and may be an effective tool for weight management in obese men. The reduction in WL and WC with T-undecanoate in more than 500 hypogonadal men [23, 34] appears to be superior when compared to data published with other drugs, in combination with lifestyle and behavioural interventions.

Our study is the first to report 6-year data of T therapy in hypogonadal obese men with T2DM, showing a progressive and sustainable reduction of HbA_1c_ levels. This result seems to be highly favorable when compared to any other reported interventions where maintaining glycaemic control over a prolonged period of time appears extremely difficult to achieve, even with a modern generation of T2DM drugs [59, 85, 86]. Moreover, the findings that a large proportion of patients reach HbA_1c_ targets appear to be unique in comparison to other studies reported in the literature. Most testosterone studies in men with T2DM and/or metabolic syndrome have been performed over a relatively short period of time. Hackett et al. [35], in their 18-month study, found the marked decrease in HbA_1c_ during the last 12 months of their study [35], indicating that these effects may take some time to occur.

As shown in the United Kingdom Prospective Diabetes Study (UKPDS), HbA_1c_ levels were not maintained over time even with intensive control treatment [59, 85]. Similarly, glycaemic control as assessed by fasting blood glucose and HbA_1c_ were not maintained over time with treatment with rosiglitazone, metformin, or glyburide monotherapy [60]. Similar trends were noted in HbA_1c_ after treatment with liraglutide [86]. These findings suggest that control of hyperglycemia is not maintained over time by these pharmacotherapeutic agents. In contrast, the data from our study showed that T therapy consistently lowered HbA_1c_, and the reduced HbA_1c_ and fasting blood glucose were maintained at the lower level and we did not observe any return to higher levels during the 72 months of T treatment. These findings are further supported by a recent study in which T therapy in diabetic patients was shown to reduce mortality, which could be explained in part by the positive effects of T on cardiometabolic function [87].

Because, six years ago when this study was commenced, we did not anticipate changes in diabetic parameters in response to T therapy, comedication was only assessed at baseline. The initial mean HbA_1c_ of 8.08% and the small proportion of men within HbA_1c_ targets at baseline indicated that the hyperglycemia in these patients was not very well controlled. The lack of assessment of antidiabetic treatment on the outcome of T therapy may represent a potential limitation of this study. It is important to note that a recent study by Hackett et al. [88] reported a marked reduction in HbA_1c_ as a result of T therapy, in addition to standard antidiabetic treatment. The reduction in HbA_1c_ in the cohort of poorly controlled patients was 0.41% by 6 weeks and was maintained at 18 and 30 weeks of treatment, respectively [88]. In this well-controlled study, there were no changes in antidiabetic medications. These findings are congruent with our observations.

Hypertension and obesity are common comorbidities in patients with T2DM. Thus, any pharmacotherapy that not only ameliorates hyperglycemia, but also improves blood pressure and reduces adipogenesis would be of interest. The prevalence of hypertension is higher in patients with T2DM and ~60% of patients diagnosed exhibit arterial hypertension. This is attributed to (i) hyperinsulinaemia, (ii) increased sympathetic tone, and (iii) increased renin-angiotensin-aldosterone system activity [89]. Our findings suggest that T therapy not only improves lipid profiles, but also improves hyperglycemia and blood pressure. This is a novel finding and merits further exploration.

One of the limitations of this study is the nature of the registry design. This combined two-center, open-label observational study was not a randomized controlled study and therefore may limit the scope of interpretation of the presented findings. Simply, obese and diabetic subjects were treated in a urology clinical setting. This may introduce unintended bias since many of the subjects are seeking medical treatment of various urological conditions. Another potential limitation is that we used total T levels and not free T levels, in combination with signs and symptoms, to evaluate TD (hypogonadism). An additional limitation was that the patients in these registries had a number of different comorbidities. Since our results were not foreseen when the study was designed and commenced six years ago, we did not assess the duration of T2DM, neither was a continued assessment of potential changes in comedications performed. Therefore, we did not have the possibility to assess whether there were any cases of remission of T2DM (defined as HbA_1c_ < 6.0% without antidiabetic medications for at least one year).

Another limitation is that we did not assess behavioral and lifestyle changes, that is, changes in nutritional and exercise habits. Since weight loss had not been expected, we did not consider the use of questionnaires or other methods to measure dietary changes or changes in physical activity.

In summary, the findings of this study demonstrate that long-term treatment with T-undecanoate in obese men with diabetes and TD restored physiological T levels and produced marked and significant WL and reduced WC and BMI. Further, T treatment significantly reduced fasting blood glucose and HbA_1c_ levels. Of equal importance, T therapy significantly reduced total cholesterol, LDL cholesterol, and triglycerides and increased HDL cholesterol levels and improved systolic and diastolic blood pressure. T therapy also reduced the levels of inflammatory markers, suggesting reduction in the inflammation response. These findings strongly suggest that T therapy of obese, diabetic men improves glycaemic control and lipid profiles and may prove useful in reducing the risk of CVD. These findings further suggest that long-term T treatment of diabetic obese men with TD may produce important clinical benefits in men with “diabesity.” One unique aspect of this study is that it followed up diabetic obese men with TD for a period of 6 years, which is the longest reported duration of T treatment to date. We suggest that T therapy in obese diabetic men may prove useful in management of men with “diabesity.”

## Figures and Tables

**Table 1:** Baseline characteristics and measures at the end of the observation period for 156 obese hypogonadal men with type 2 diabetes mellitus treated with testosterone undecanoate for up to 6 years.

	Baseline	Endpoint
Mean age (years)	61.17 ± 6.18	
Anthropometry
Weight (kg)	113.56 ± 11.53	97.18 ± 9.05
BMI (kg/m^2^)	36.31 ± 3.51	31.19 ± 2.6
Normal weight (proportion)	0	1 (0.6%)
Overweight (proportion)	0	48 (30.8%)
Obese (proportion)	156 (100%)	107 (68.6%)
Waist circumference (cm)	114.0 ± 8.69	102.52 ± 7.93
Normal (<94 cm)	1 (0.6%)	7 (4.5%)
Increased (≥ 94 cm)	155 (99.4%)	149 (95.5%)
Diabetic parameters		
Fasting glucose (mg/dL)	128.37 ± 31.63	101.55 ± 17.02
Fasting glucose (mmol/L)	7.06 ± 1.74	5.59 ± 0.94
HbA1c	8.08 ± 0.9	6.14 ± 0.71
Other metabolic parameters
Total cholesterol (mg/dL)	294.85 ± 47.65	194.38 ± 16.79
Total cholesterol (mmol/L)	7.64 ± 1.23	5.03 ± 0.43
HDL cholesterol (mg/dL)	56.63 ± 19.8	67.88 ± 17.34
HDL cholesterol (mmol/L)	1.47 ± 0.51	1.76 ± 0.45
LDL cholesterol (mg/dL)	177.84 ± 28.34	125.47 ± 33.05
LDL cholesterol (mmol/L)	4.61 ± 0.73	3.25 ± 0.86
Triglycerides (mg/dL)	297.05 ± 71.25	197.9 ± 23.24
Triglycerides (mmol/L)	3.36 ± 0.81	2.24 ± 0.26
Systolic blood pressure (mmHg)	157.03 ± 15.46	134.61 ± 10.21
Diastolic blood pressure (mmHg)	93.89 ± 11.67	79.0 ± 5.57
Pulse pressure	63.07 ± 10.7	55.61 ± 8.62
Total cholesterol : HDL ratio	6.02 ± 2.97	3.05 ± 0.78
LDL : HDL ratio	3.61 ± 1.71	1.88 ± 0.41
CRP (mg/dL)	3.16 ± 4.12	0.72 ± 0.56
Liver enzymes		
AST	35.55 ± 13.28	23.94 ± 8.77
ALT	39.04 ± 19.38	26.08 ± 14.42
Testosterone		
Testosterone (nmol/L)	8.9 ± 1.99	17.36 ± 1.92

**Table 2:** Comorbidities and concomitant medications at baseline for 156 obese hypogonadal men with type 2 diabetes mellitus treated with testosterone undecanoate for up to 6 years.

Comorbidities	
Hypertension	153 (99.4%)
Type 2 diabetes	156 (100%)
Dyslipidaemia	156 (100%)
Coronary artery disease	37 (23.7%)
Myocardial infarction	19 (12.2%)
Concomitant medications for diabetes at baseline	
Metformin	36 (23.1%)
Insulin	25 (16%)
Sulfonylurea	8 (5.1%)
Acarbose	2 (1.3%)
Antidiabetic drugs not further specified	51 (32.7%)
Total antidiabetic drugs	122 (78.2%)
No diabetes drugs (including 2 patients who were “prescribed” a diet)	29 (18.6%)
No information available	9 (5.8%)
Other concomitant medication categories at baseline	
Antihypertensives	123 (78.8%)
Lipid-lowering drugs	99 (63.5%)

**Table 3:** Changes in anthropometric and metabolic parameters in subgroups of obese hypogonadal men with type 2 diabetes and total testosterone <8 nmol/L versus total testosterone >8 nmol/L and <12 nmol/L after treatment with testosterone undecanoate for up to 6 years.

	T < 8 nmol/L (*n* = 53)	T > 8 nmol/L (*n* = 103)
Mean age (years)	61.43 ± 7.78	60.96 ± 5.24
Anthropometry		
Decrease in weight (kg)	16.05 ± 1.01	18.42 ± 0.71
Weight reduction from baseline (%)	13.75 ± 0.84	15.88 ± 0.58
Decrease in BMI (kg/m^2^)	5.06 ± 0.32	5.94 ± 0.22
Decrease in waist circumference (cm)	12.84 ± 0.64	10.77 ± 0.36
Diabetic parameters		
Decrease in fasting glucose (mg/dL)	32.31 ± 5.19	24.4 ± 2.42
Decrease in HbA1c (%)	1.98 ± 0.09	1.9 ± 0.07
Other metabolic parameters		
Decrease in total cholesterol (mg/dL)	94.99 ± 8.72	106.21 ± 4.04
Increase in HDL cholesterol (mg/dL)	14.87 ± 3.08	10.78 ± 1.3
Decrease in LDL cholesterol (mg/dL)	51.28 ± 5.29	44.07 ± 2.83
Decrease in triglycerides (mg/dL)	107.73 ± 12.23	98.55 ± 5.64
Decrease in systolic blood pressure (mmHg)	22.11 ± 1.56	23.57 ± 0.97
Decrease in diastolic blood pressure (mmHg)	9.21 ± 1.41	18.29 ± 0.88
Testosterone		
Increase in testosterone (nmol/L)	11.45 ± 0.54	6.37 ± 0.37
